# Deconstruction: the qualitative methodology for the analysis of sustainability assessment tools of agri-system

**DOI:** 10.1016/j.mex.2018.06.003

**Published:** 2018-06-12

**Authors:** Lorenzo Bonisoli, Emilio Galdeano-Gómez, Laura Piedra-Muñoz

**Affiliations:** a Universidad Técnica de Machala (Unidad Académica de Ciencias Empresariales), Ecuador; b University of Almería (Agrifood Campus of International Excellence, ceiA3), Spain

**Keywords:** Deconstruction, Agriculture sustainability, Sustainability assessment tools, Sustainability indicators

## Abstract

As sustainability is a philosophical concept, the evaluation of sustainability of an agri-system is underpinned by a philosophical understanding. Deconstruction is the qualitative methodology derived from philosophical science that allows to show what is hidden, to reveal the implicit meaning of a sustainability assessment tool.

• Qualitative methodology of analysis.
• Applicable to all kind of qualitative analysis.
• Suitable for review article.

## Graphical abstract

**Figure:**
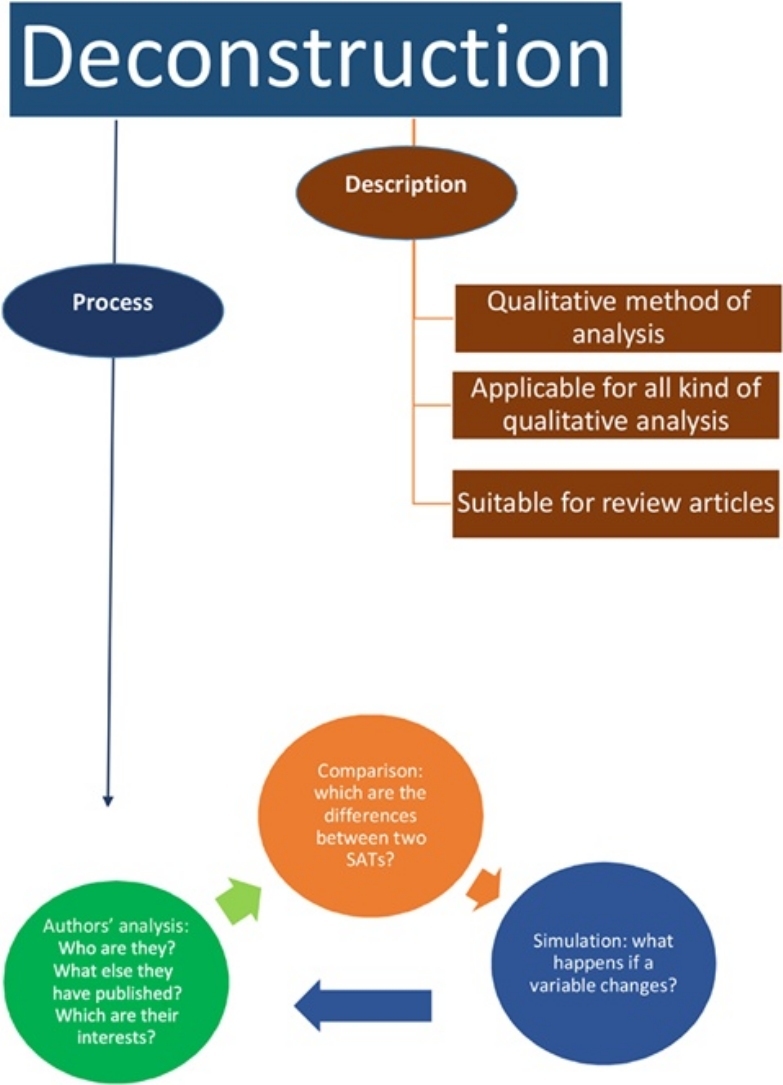

**Specifications Table**

**Table:** 

Subject area	*Select one of the following subject areas*: • *Environmental Science*
More specific subject area	*Agriculture sustainability*
Method name	*Deconstruction*
Name and reference of original method	Derrida, J. (1974). *Of Grammatology*, trans. Gayatri Spivak. Baltimore: The Johns Hopkins University Press. Derrida, J. (1978). *Writing and Difference*, trans. Alan Bass. Chicago: University of Chicago Press.
Resource availability	*No applicable*

## Background

In recent years, in the academic arena the application of sustainability principles to the agricultural sector has become a crucial subject of study. However, despite a general accord on its relevance, the concept of sustainability lacks a consensus on its definition and in the methodology for its evaluation [1].

Regarding this last point, practitioners and analysts have developed in the last years several sustainability assessment tools (SAT) that employ a group of indicators to evaluate the sustainability of an agri-system [2].

Studies on SAT showed that these instruments can vary on different issues [3], for example the end-users they are addressed to (for instance they may be thought for practitioners, for policy makers or for academics), the aim they are designed to and the concept of sustainability underpinning the instrument.

In the analysis of the literature it is possible to find several studies about SAT [4] but just a minority of them discuss the evaluation process in depth while the great majority focuses on applications and results. In addition, since every SAT is underpinned by a precise concept of sustainability [5], the evaluation process and results are implicitly shaped by this underlying philosophical concept. For this reason it is difficult for practitioners to understand the reason why a SAT is used by other analysts and which SAT best fits the requirements of a specific agri-system; and the need of a methodology that allows to show the philosophical understanding.

In general it is possible to state that in the literature a precise methodology for qualitative analysis is missed. This study aims to introduce a methodology for the qualitative evaluation derived from the philosophical sciences that allows practitioners and analysts to fully understand the SAT in order to choose the most suitable for a given agri-system.

Deconstruction is a methodology firstly developed by the French philosopher Jacques Derrida [6,7] and originally applied to philosophical analysis. Deconstruction is a qualitative methodology that allows researchers and practitioners to analyse SAT in order to choose the most appropriate for the evaluation’s purpose. Deconstruction is not only interested in the results of a sustainability evaluation, but it focuses in particular in the criteria for the indicators inclusion in the SAT and in its methodology [8].

## Method details

This methodology relies on three basic assumptions:

First, in a SAT nothing is casual. This methodology considers that all conceptual tools are built using a precise logic that is functional to SAT purpose.

Second, the logic behind the SAT is underpinned by a precise philosophical understanding.

Third, there is not a “best” philosophical view, thus the purpose of the analyst is not to judge the different concepts of sustainability but to reveal the concept behind the instrument.

**Chart 1:**
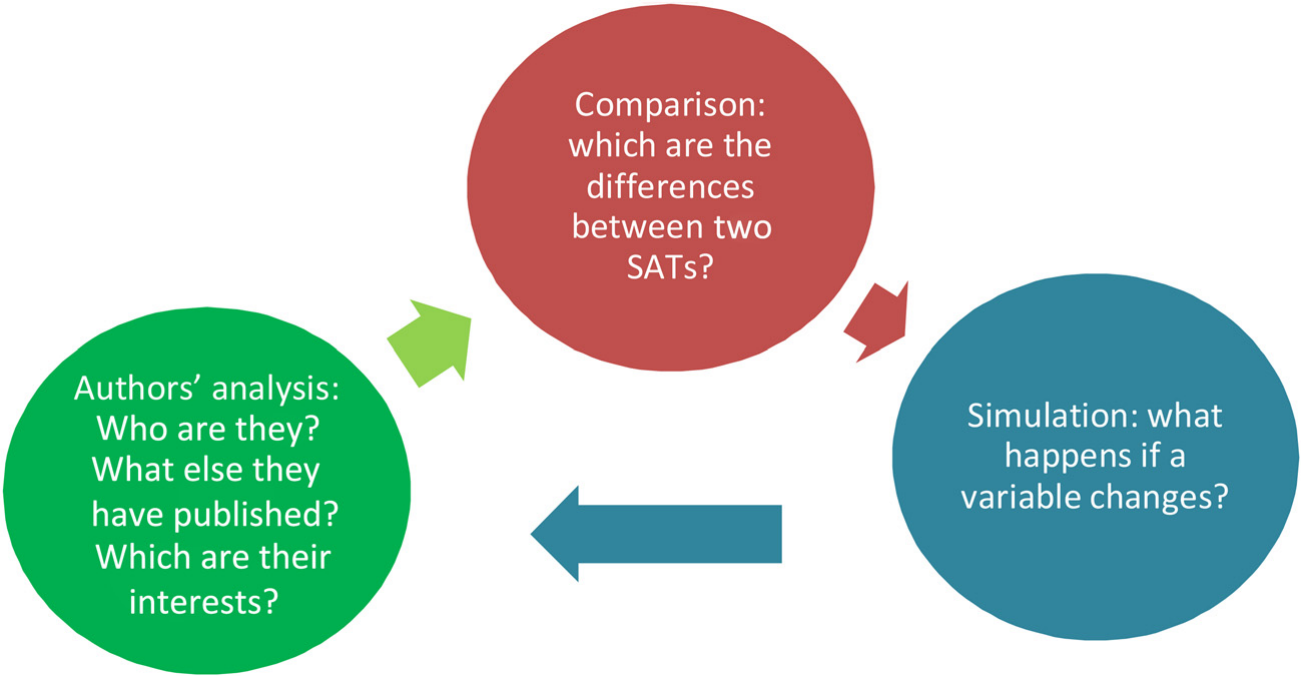
Deconstruction process.

Deconstruction has not a formal set of steps for its application but can uses different tactics. A possible process could be (see Chart 1):

### Comparison

A first tactic is to compare different SAT in order to find the differences and to interrogate the consequence those differences lead. In this way SAT can be compared regarding the indicators they use, for example, the number of indicators, the dimensions they cover, if they are qualitative or quantitative. Moreover SAT can be compare in the aggregate approach employed in the evaluation and the methods to show the results to end-users. Finally, SAT may be compared in the way stakeholders are involved in the process or if it is possible for farmer to enhance the sustainability of the enterprise using the results of the assessment.

### Simulation

Other tactic is to apply the SAT with extreme and fictional input in order to analyse the possible results. For example practitioners can imagine a situation in which an evident unsustainable problem occurs (for example: unfair price negotiation or raising unemployment rate) to check whether the SAT identifies or to which extent the results are affected by the problem.

### Author analysis

This tactic considers to investigate other article of the same authors to check if there are relationships among different studies. It is possible that the same author who in an article presents a new SAT, in another study is claiming the need for a certification of sustainable product similar to the certification of organic product; thus, the aim of the SAT is probably to be the instrument to evaluate a future certification in a project of a certification business.

## Example: MESMIS and MMF

MESMIS, Framework for Assessing the Sustainability of Natural Resource Management Systems [9,10], and MMF, Multiscale Methodological Framework [11], are two similar SATs that are usually treated together because of the similar structure and the fact that professor López-Ridaura is among the main authors of both SATs.

In this case the first deconstruction approach to apply is the comparison since we need to understand the reason why a professor who is one of the leading developers of a SAT elaborates a similar but alternative SAT just three years after the introduction of the first SAT in the global academic debate.

Both SATs rely in a set of sustainability attributes that underpin the sustainability evaluation process. Those attributes for MESMIS are: productivity, stability, resilience, reliability, adaptability, equity and self-reliance. MMF shares the same attributes with the exception of equity and self-reliance with the following justification: “Other attributes such as empowerment, equity and adaptability have explicitly been included in attempts to integrate the social dimension in the analysis, rather than as basic attributes of sustainable systems which are independent of the disciplinary approach” ([11], p. 54). The authors do not mention why self-reliance is not included, however, they refers that some of the excluded attributes have “disciplinary bias” ([11], p. 54), so it is possible that this MESMIS attribute has this flaw.

From the attributes, both SATs develop a set of indicators. This process in MESMIS is carried out through the identification of system critical points, such as the sustainability issues of the analysed system that are related to the attributes. On the other hand, in MMF this process is developed with the interaction between analysists and key stakeholders.

These two kind of differences between MESMIS and MMF (i.e. the exclusion of two sustainability attributes and different process for indicators identification) leads to a relevant variance in the analysis. In fact, while MESMIS sets indicators of equity for each sustainability dimension, MMF may completely underrate not only equity as a sustainability attribute but the social dimension as a whole.

The evidence of that is the set of general indicators shown by the case study presented by MMF in which no social indicators at all are applied to evaluate the sustainability at the farm scale of the agriculture system in Purhepecha Region of Michoacán, Mexico ([11], p. 65).

In conclusion, this brief comparison shows how the desire of MMF to be more close to stakeholders´ needs may lead to an underestimation of key sustainability issues such as social equity.
